# MicroRNA 433 regulates nonsense-mediated mRNA decay by targeting *SMG5* mRNA

**DOI:** 10.1186/s12867-016-0070-z

**Published:** 2016-07-29

**Authors:** Yi Jin, Fang Zhang, Zhenfa Ma, Zhuqing Ren

**Affiliations:** 1 Key Laboratory of Swine Genetics and Breeding of Ministry of Agriculture & Key Laboratory of Agriculture Animal Genetics, Breeding and Reproduction of Ministry of Education, College of Animal Science, Huazhong Agricultural University, Wuhan, 430070 Hubei People’s Republic of China; 2 The Cooperative Innovation Center for Sustainable Pig Production, Huazhong Agricultural University, Wuhan, 430070 Hubei People’s Republic of China

**Keywords:** miR-433, *SMG5*, NMD

## Abstract

**Background:**

Nonsense-mediated mRNA decay (NMD) is a RNA quality surveillance system for eukaryotes. It prevents cells from generating deleterious truncated proteins by degrading abnormal mRNAs that harbor premature termination codon (PTC). However, little is known about the molecular regulation mechanism underlying the inhibition of NMD by microRNAs.

**Results:**

The present study demonstrated that miR-433 was involved in NMD pathway via negatively regulating *SMG5*. We provided evidence that (1) overexpression of miR-433 significantly suppressed the expression of *SMG5* (P < 0.05); (2) Both mRNA and protein expression levels of *TBL2* and *GADD45B*, substrates of NMD, were increased when *SMG5* was suppressed by siRNA; (3) Expression of *SMG5*, *TBL2* and *GADD45B* were significantly increased by miR-433 inhibitor (P < 0.05). These results together illustrated that miR-433 regulated NMD by targeting *SMG5* mRNA.

**Conclusions:**

Our study highlights that miR-433 represses nonsense mediated mRNA decay. The miR-433 targets 3’-UTR of *SMG5* and represses the expression of *SMG5*, whereas NMD activity is decreased when SMG5 is decreased. This discovery provides evidence for microRNA/NMD regulatory mechanism.

## Background

Nonsense-mediated mRNA decay (NMD) recognizes and degrades mRNAs that contain premature termination codon (PTC). This surveillance mechanism could prevent from generating deleterious truncated proteins [1, 2]. UPF1 is an essential factor for NMD, which can recognize abnormal translation termination, and activate NMD [3]. Previous studies have indicated that suppressor with morphogenetic effect on genitalia 5 (SMG5) was involved in de-phosphorylation of UPF1 [4, 5]. SMG5 and 7 shared a conserved 14-3-3 like domain that could recognize the phosphorylated UPF1 [6]. When UPF1 was de-phosphorylated, *SMG5* and *7* were recruited to the P-bodies [7]. Phosphorylated serine 1096 of UPF1 could bind the heterodimer formed by SMG5–7 [8]. In addition this heterodimer cold increase affinity combining to UPF1 [9]. SMG5–7 and protein phosphatase 2A (PP2A) plays an important role in the recycling for UPF1 [10, 11]. Furthermore, SMG5–7 interacts with deadenylases CCR4-NOT complex [12], and SMG5 was associated to the decapping enhancer Prorich nuclear receptor co-activator 2 (PNRC2) recruiting the general decapping complex [13]. As the consequence, it is very important to investigate the expression and regulation of SMG5 for further understanding of NMD. One recent study indicated that DEAD box protein Ddx5 regulated *SMG5* expression [14]. However, few studies have reported that microRNAs could regulate *SMG5* expression. Here we demonstrated that a microRNA, miR-433, regulated *SMG5* expression. Shih et al indicated that miR-433 was associated to poorer survival in ovarian cancer [15]. MiR-433 expression was altered in gastric cancer [16], lung dysplasia and myeloproliferative neoplasms [17, 18]. MiR-433 could also regulate the expression of *SFRP2*, *GRB2*, *CREB1*, and *HDAC6*, all of which were closely associated to cancer [19–23].

Two recent studies reported that miR-128, miR-125a, and miR-125b regulated NMD through silencing *UPF1* and *SMG1* [24, 25]. In present study, we demonstrated that miR-433 repressed the expression of *SMG5*, thereby suppressed NMD activity. Additionally, there may be a miR-433/NMD regulatory circuit in eukaryotic cells.

## Results

### MiRNA-433 targets 3′-UTR of SMG5

**Fig. 1:**
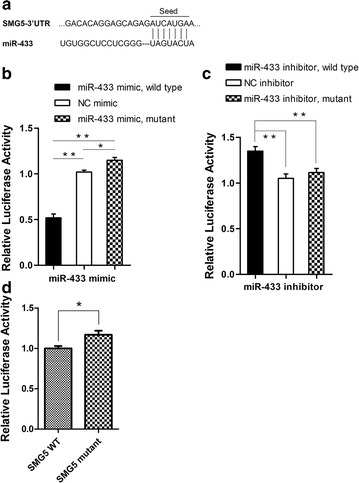
MiR-433 targets the 3′-UTR of *SMG5*. **a** The predicated seed sequence in 3’-UTR of *SMG5* by targetscan (http://www.targetscan.org/). **b** Luciferase expression level in HEK 293T cells transfected for 24 h with miR-433 mimic or a negative-control microRNA mimic. **c** Luciferase expression in Hela cells transfected for 48 h with miR-433 inhibitor or a negative-control inhibitor. **d** Luciferase expression level of the *SMG5* WT vs* SMG5* mutant in HEK 293T cells transfected for 24 h with a negative-control microRNA mimic (NC). *P < 0.05, and ** means the difference is significant, P < 0.01

We selected the possible miRNAs targeting *SMG5* through miRNA algorithm TargetScan (http://www.targetscan.org/), miRanda (http://www.microrna.org/microrna/home.do), RNAhydrid (http://www.bibiserv.techfak.uni-bielefeld.de/rnahybrid/), and FINDTAR (http://www.bio.sz.tsinghua.edu.cn/). The prediction of all the softwares indicates that miR-433 targets *SMG5* (Fig. 1a). The 3′-UTR of *SMG5* holds a sequence motif (AUCAUGA) that is identical to the seed sequence UAGUACU (UAGUACU) of *miR*-*433* (Fig. 1a). We performed the dual-luciferase assay to investigate miR-433 targeting *SMG5*. The wild report gene vector pmirGLO-SMG5-WT and miR-433 mimic were co-transfected into HEK-293T cells. Then we detected the normalized luciferase values, which showed 50 % reduction compared with controls (P < 0.01, Fig. 1b). However, this regulation was abrogated when a four-nucleotide mutation (AUGA–UACU) was introduced in the miR-433 seed sequence in the 3′-UTR of *SMG5* (Fig. 1b). Additionally, the expression level of luciferase in SMG5 mutant was increased compared to SMG5 WT in the presence of NC (Fig. 1d). Furthermore, the dual-luciferase vectors and miR-433 inhibitor were co-transfected into Hela cells. The result showed *SMG5* expression was increased (Fig. 1c). This regulation was also abrogated when the pmirGLO-SMG5-MUT was transfected (Fig. 1c). In general, the results together showed that miR-433 targets 3′-UTR of *SMG5* directly.

### The suppression of SMG5 repressed NMD activity

**Fig. 2:**
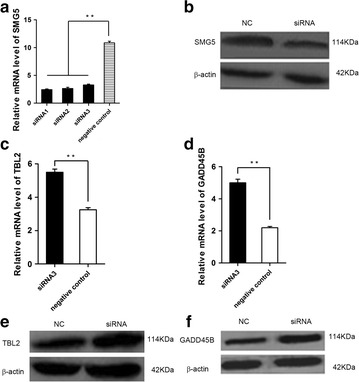
Knockdown *SMG5* expression repressed NMD activity. **a** Quantitative polymerase chain reaction (qPCR) analysis of *SMG5* mRNA expression in C2C12 cells transfected for 24 h with either three RNA interference fragments or negative control, positive control and Mock respectively. **b** Western Blot analysis of endogenous *SMG5* protein levels in C2C12 cells 24 h after transfection with the RNA interference fragment or a negative control fragment. *β*-actin was used as the internal control. **c**–**f** showed the qPCR and western blot analysis of NMD substrates,* TBL2 *and *GADD45B*, mRNA and protein level in C2C12 cells 24 h after transfection with the RNA interference fragment or a negative control fragment. *β*-actin was used as the internal control. *P < 0.05; **P < 0.01

The previous studies have indicated that SMG5 was an important NMD factor, so we are going to suppresse *SMG5* expression by RNAi to confirm this function. The siRNA was transfected into C2C12 cells for 24–48 h. The results showed that *SMG5* was decreased in both mRNA and protein expression level (Fig. 2a, b). According to the NMD mechanism, if NMD activity reduced, the expression of NMD substrates would be reduced. The NMD activity thereby was indicated by the expression level of NMD substrates, *TBL2* and *GADD45B*. We detected the expression level of *TBL2* and *GADD45* and found that both of them were increased (P < 0.05, Fig. 2c–f). Therefore the RNAi experiment indicated that suppression of SMG5 repressed NMD activity.

### MiR-433 repressed SMG5 expression

**Fig. 3:**
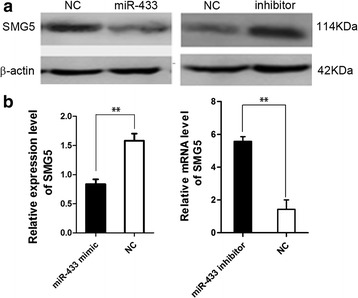
MiR-433 reduced *SMG5* protein and mRNA level. **a** Western Blot analysis of endogenous *SMG5* protein levels in BHK cells 48 h after transfection with mature miR-433 mimic or a random negative-control miRNA mimic (in C2C12 cells 48 h after transfection with miR-433 inhibitor or a random negative-control). *β*-actin was used as the internal control. **b** Quantitative polymerase chain reaction (qPCR) analysis of BHK cells transfected for 48 h with the miR-433 mimic or inhibitors (in C2C12 cells). Shown are the results from three independent experiments, normalized to *β*-actin mRNA levels. **P < 0.01

When we transfected miR-433 mimic into BHK cells, the expression level of *SMG5* was decreased, (P < 0.01, Fig. 3). Furthermore, the expression level of *SMG5 *was increased significantly when the miR-433 inhibitor was transfected into the BHK cells (P < 0.01, Fig. 3). As the consequence, the *SMG5* expression was down-regulated by miR-433.

### MiR-433 repressed NMD activity

**Fig. 4:**
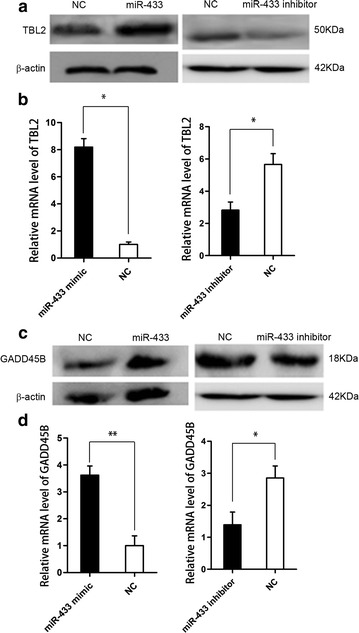
**a**, **c** Western Blot analysis of endogenous NMD substrates, *TBL2* and *GADD45B* protein levels in cells 24 h after transfection with the miR-433 mimic (in BHK cells) or miR-433 inhibitor (in C2C12 cells) or a negative control miRNA mimic. *β*-actin was used as the internal control. **b**, **d** QPCR analysis of two NMD substrates, *TBL2* and *GADD45B* relative mRNA expression level in cells 24 h after transfection with the miR-433 mimic (in BHK cells) or miR-433 inhibitor (in C2C12 cells) or a negative control miRNA mimic. *P < 0.05; **P < 0.01

Since we have demonstrated that miR-433 repressed *SMG5* expression and suppression of *SMG5* repressed NMD activity, miR-433 would repress NMD activity. In this section, the results showed that the NMD substrates *TBL2* and *GADD45B* were increased in BHK cells when transfected with miR-433 mimic (P < 0.05, Fig. 4). In addition, we chose another cell line (C2C12) to transfect miR-433 inhibitor. The expression level of *TBL2* and *GADD45B* were also decreased by miR-433 inhibitor (P < 0.05, Fig. 4).

## Discussion

Nonsense-mediated mRNA decay, a surveillance system, which recognizes mRNAs with translation termination codons positioned in abnormal contexts and degrades aberrant mRNAs, scrutinizes mRNA quality in all mammalian cells [26]. In this intricate process, a set of NMD factors are influential to the recognition and degradation of aberrant mRNAs. *UPF1*, the master regulator of NMD, is considered to determine the NMD process [27–29]. The ATPase and helicase activities as well as the phosphorylation of N- and C- terminal domains bestow on UPF1 ability that recognizes premature termination codon containing mRNAs selectively [30–33]. The phosphorylation and de-phosphorylation of UPF1 were very important for NMD. SMG5, an important NMD factor, played an important role in UPF1 de-phosphorylation process. As our results showed, NMD activity was repressed when the expression of *SMG5* was suppressed. So the regulation for *SMG5* could affect NMD activity. We selected a microRNA, miR-433, which targets 3′-UTR of *SMG5* by prediction. The regulation of miR-433 targeted *SMG5* was detected by dual-luciferase report assay. The result indicated that *SMG5* expression was reduced by 50 % when we transfected with miR-433 mimic. It has demonstrated that the NMD activity was repressed when *SMG5* was suppressed (Fig. 3). Hence, miRNA-433 repressed NMD activity by suppressing *SMG5* expression.

As a conserved mRNA surveillance system in eukaryotic cells, NMD is crucial for many physiological processes, and these crucial function of NMD have been published by many researches. Numbers of NMD factors were important for embryo development. When *UPF1*, *UPF2*, *SMG1* or *SMG6* was knockout or knockdown technologically, all mice died during an early embryonic stage [34–37]. A study implied that NMD has the potency that affects cell type diversification [34]. NMD activity was identified to be reduced in neuronal stem cells differentiation process. And this regulation of NMD activity is essential for nervous system development [38]. Additionally, NMD also regulates neural development related genes [39, 40]. Here, our experiment indicated that miR-433 repressed *SMG5* expression, and therefore suppressed NMD activity. This result indicated a circuit regulation of miR-433 and NMD. Hence, miR-433 could participate in the process regulated by NMD. For the circuit regulation, previous study identified miR-128 repressed NMD by *UPF1* and *MLN51* [24]. MiR-125 and 128 were involved in many nervous system disorder diseases such as autism [41], prion-induced neuron degeneration [42], Huntington’s disease [43], Parkinson’s disease [44], as well as Alzheimer’s disease [45]. Some studies indicated that NMD played an important role in brain development and embryonic development [46, 47], and three essential embryonic brain vesicles formation disrupted by knockout of *SMG1* [48]. Therefore, we assumed that there may be a miR-433/NMD regulatory circuit in early embryo development, especially in early nervous and brain development. This study would provide evidence to make clear the microRNA/NMD regulatory mechanism and give advice to neurological disease and related cancers therapies.

## Conclusion

It is well known that nonsense mediated mRNA decay is an important mRNA quality surveillance system. However, the microRNA/NMD regulatory mechanism is not fully clear. Our study claim that NMD activity is repressed by a microRNA, miR-433. The miR-433 targets 3′UTR of *SMG5* and decreases the expression of *SMG5*. NMD activity is suppressed when expression of *SMG5* is reduced. This discovery provides evidence for microRNA/NMD regulatory mechanism and therapeutic advice for NMD-related diseases.

## Methods

### Cell culture

The cell lines, HEK-293T, Hela, BHK-21, and C2C12, were purchased from China for type culture collection (CCTCC). Cells expect for Hela were maintained in Dulbecco’s modified Eagle’s medium (DMEM, Hyclone, Logan, Utah, USA) supplemented with 10 % fetal bovine serum (FBS, Hyclone, Logan, Utah, USA) at 37 °C in a humidified atmosphere of 5 % CO_2_. Hela cells were maintained in DMEM (Hyclone, Logan, Utah, USA) containing 20 % FBS (Hyclone, Logan, Utah, USA) at 37 °C in a humidified atmosphere of 5 % CO_2_. Medium was changed twice weekly until the fibroblasts migrated out to cover the dishes. Passage 6–9 cells were used for all experiments.

### Isolation of 3′-UTR sequences of SMG5

C2C12 genome DNA was used as template to amplify 3′-UTR of SMG5. To isolate the 464-bp 3′-UTR of 2.6-kb genome, the following primer pairs were used: SMG5-3′UTR-F and SMG5-3′UTR-R [5′-cgagctcCTGTACTGGATAAGGGGTGCC-3′ and 5′-ccgctcgagCAACTGTTCCCTGGTTTTCCC, with uppercase bases corresponding to the mouse 2.6-kb SMG5 genome and lowercase bases indicating 5′ extensions with restriction enzyme sites (underline) for *Sac*I and *Xho*I, respectively]. The protocol was as follows: pre-denaturation at 94 °C for 2 min, denaturation at 94 °C for 30 s, annealing at 64 °C for 30 s, elongation at 72 °C for 30 s per 1 kb, thirty-five cycles, elongation at 72 °C for 2 min, and end. PCR products were investigated by 1–2 % agarose gel electrophoresis. The restriction enzyme, *Sac*I and *Xho*I were purchased from Fermentas (Thermo Fisher Scientific, Rockford, IL, USA).

### Wild type report gene constructs

The *2.2.* PCR product was purified and cloned into the *Sac*I and *Xho*I sites of pmirGLO dual-luciferase miRNA target expression vector (Promega, Madison, WI, USA). The enzyme digestion system contained 10× buffer 4 μL, *Sac*I 4 μL, *Xho*I 4 μL, pmirGLO vector 12 μL, and the ddH_2_O up to 40 μL. The enzyme digestion system was incubated at 37 °C for 2 h. The T_4_ DNA ligase (TaKaRa) interaction system contained enzyme digestion fragment 3 μL, digested pmirGLO plasmid 2 μL, Solution I 5 μL. The system was incubated at 16 °C for 2 h. The accuracy of inserted 3′-UTR of *SMG5* were confirmed by sequencing. Then the plasmid was isolated and purified using E.Z.N.A. ^®^ endo-free plasmid mini kit II (OMEGA, Bio-Tek, Norcross, GA, USA). The constructed wild type report gene recombinants named pmirGLO-SMG5-WT was stored at −20 °C.

### Introducing four-nucleotide mutagenesis into *SMG5* 3′-UTR

Two primer pairs, SMG5-3′UTR-F (5′-cgagctcCTGTACTGGATAAGGGGTGCC-3′) and SMG5-3′UTR-R (5′-ccgctcgagCAACTGTTCCCTGGTTTTCCC), SMG5-3′UTR-MF (5′-AGCAGAGATC**TACT**ATACTCAG-3′) and SMG5-3′UTR-MR (5′-GCCCCTGAGTAT**AGTA**GATCTCTG-3′), were designed (uppercase bases corresponding to the mouse 2.6-kb *SMG5* genome and lowercase bases indicating 5′ extensions with restriction enzyme sites [underline] for *Sac*I and *Xho*I, respectively. And the bold bases mean four-nucleotide mutation sites of *SMG5* 3′-UTR). The former was used to construct pmirGLO-SMG5-WT, whereas the latter to amplify the mutation sites and its upstream sequence (production I), and the mutation sites and its downstream sequence (production II) respectively, the pmirGLO-SMG5-WT plasmid was the template. Then using fusion PCR combined these two products. The PCR system contained 2× taq PCR mix 11 μL, recovered production I 2 μL, recovered production II 2 μL, ddH_2_O 10 μL. PCR conditions: an initial denaturation at 95 °C for 4 min, followed by 30 cycles of 95 °C for 30 s, 40 °C for 30 s, 45 °C for 5 s, 50 °C for 5 s, 55 °C for 5 s, 60 °C for 5 s and 72 °C for 30 s, with a final extension of 5 min at 72 °C. PCR products were visible after electrophoresis of 25-μL reaction using an agarose gel (1.5 %). Then the fusion PCR production was recovered and used as a template.

### Mutant report gene constructs

The 464 bp mutant *SMG5* 3′-UTR was amplified, purified, and cloned into the *Sac*I and *Xho*I sites of pmirGLO dual-luciferase miRNA target expression vector (Promega, Madison, WI). The enzyme digestion system contained 10× buffer 4 μL, *Sac*I 4 μL, *Xho*I 4 μL, pmirGLO vector 12 μL, and ddH_2_O up to 40 μL. The enzyme digestion system was incubated at 37 °C for 2 h. The T_4_ DNA ligase (TaKaRa) interaction system contained enzyme digestion fragment 3 μL, digested pmirGLO plasmid 2 μL, Solution I 5 μL. This system was incubated at 16 °C for 2 h. The accuracy of inserted SMG5 3′-UTR was confirmed by sequencing. Then the plasmid was isolated and purified using E.Z.N.A. ^®^ endo-free plasmid mini kit II (OMEGA, Bio-Tek, Norcross, GA, USA). The mutant report gene vector,named pmirGLO-SMG5-MUT conserved at −20 °C.

### Cell transfection and dual-luciferase assay

HEK-293T cells were seeded onto 24-well plates at 30–40 % confluence. The next day, medium was changed to Opti-MEM I Reduced Serum Medium (Hyclone, Logan, Utah, USA), and cells were transfected with pmirGLO-SMG5-WT constructs (200 ng per well). After 4–6 h, medium was replaced by MEM containing 10 % FBS (Hyclone, Logan, Utah, USA). Transfected cells were washed twice with cold PBS (Hyclone, Logan, Utah, USA), lysed using 1× passive lysis buffer (Promega) for 30 min, and assayed for firefly and Renilla luciferase activities by the dual-luciferase assay (Promega) in a PerkimELmer 2030 Microplate Reader (Lenovo, China) according to the manufacturer’s instructions. At least three independent experiments were performed for each assay, each time with a minimum of n = 4. For co-transfection, cells were grown on 24-well plates. For each well, a transfection mixture (200 μL) consisting of 0.2-μg reporter construct, 1.25 μL miR-433 mimic or negative control, and 2 μL Lipofectamine 2000 Reagent (Invitrogen) was prepared. Opti-MEM I Reduced Serum Medium was used as diluent. Hella cells were treated as same as the HEK-293T, but the medium was MEM containing 20 % FBS.

### RNA isolation and mRNA/miRNA quantitation

The RNA isolation reagent was TRizol^®^ Reagent (Invitrogen, Carlsbad, CA, USA). RNA was quantified spectroscopically (ND-1000 Spectrophotometer; NanoDrop, Wilmington, DE, USA), and integrity was assessed by agarose gel electrophoresis. According to the manufacturer’s protocol, RNA was reverse-transcribed using RNA PCR kit (Takara Bio Inc.). The gene expression was investigated by RT-PCR. Primers for RT-PCR were as follows: *SMG5*-qF (5′-TACCTCATCCCTGACACCCA-3′) and *SMG5*-qR (5′-GCCCCTGGCTGTTCTTTCT-3′), miR-433-qF (CTGGTAGGATCATGATGGGAT) and miR-433-qR (TCAACTGGTGTCGTGGAGT), *U6*-qF (CTGGTAGGGTGCTCGCTTCGGCAG) and *U6*-qR (CAACTGGTGTCGTGGAGTCGGC), *TBL2*-qF (5′-AAGTATCTGGCCACCTGTGC-3′) and *TBL2*-qR (5′-GGCCAGCCAAACAATGAAGG-3′), *GADD45B*-qF (5′-AAGGCGGCCAAACTGATGAAT-3′) and *GADD45B*-qR (5′-ATTGTCGCAGCAGAACGACT-3′), *β*-*actin*-F (5′-GCCTCACTGTCCACCTTCCA-3′) and *β*-*actin*-R (5′-AGCCATGCCAATGTTGTCTCTT-3′). All the primer pairs for each gene were used for gene-specific amplification. The expression of *β*-*actin* was the internal control. For quantification, the comparative threshold cycle method was used to assess relative changes in mRNA levels [49].

### MicroRNA overexpression assay

MiR-433 mimic and inhibitor were purchased from GenePharma (shanghai, China). MiR-433 mimic could enhance endogenous miR-433 function, and miR-433 inhibitor could repress the endogenous miR-433 function. HK-21 cells and C2C12 cells were seeded onto 6-well plates in DMEM supplemented with 10 % FBS at 37 °C in a humidified atmosphere of 5 % CO_2_. The next day, when the cells confluence get 70–80 %, the medium was changed to Opti-MEM I Reduced Serum Medium (Hyclone, Logan, Utah, USA), and cells were transfected with 10 μL miRNA mimic (0.02 nmol/μL) using Lipofectamine 2000 Reagent (Invitrogen). After 4–6 h, medium was replaced by MEM containing 10 % FBS (Hyclone, Logan, Utah, USA). Transfected cells were washed twice with cold PBS (Hyclone, Logan, Utah, USA). At least three independent experiments were performed for each assay, each time with a minimum of n = 4. Opti-MEM I Reduced Serum Medium was used as diluent. The mimic was added fluorescent tag so that it could be detected under fluorescence microscope to calculate the transfection efficiency.

### Small interfering RNA design and cell transfection

The *musSMG5* mRNA sequence and internal control (*β-*actin) mRNA sequences were obtained from NCBI database (http://www.ncbi.nlm.nih.gov/). The siRNAs were purchased from Genpharma (Inc. Shanghai, China). The sequences if siRNAs were as follows: siRNA1 5′-GCCGCUUCAUCAUCAUCAUTT-3′ for sense and 5′-AUGAUGAUGAUGAAGCGGCTT-3′ for antisense; siRNA2 5′-GGAGUGUGAAAGUGGAUAUTT-3′ for sense and 5′-AUAUCCACUUUCACACUCCTT-3′ for antisense; siRNA3 5′-GCAGGCAGCAAGUAUUACATT-3′ for sense and 5′-UGUAAUACUUGCUGCCUGCTT-3′ for antisense; NC (negative control) 5′-UUCUCCGAACGUGUCACGUTT-3′ for sense and 5′-ACGUGACACGUUCGGAGAATT-3′ for antisense. C2C12 cells were incubated in 5 % CO2 at 37 °C in DMEM (Hyclone, Logan, Utah, USA) supplemented with 10 % FBS (Hyclone, Logan, Utah, USA). One day before transfection, 10^5^ cells were plated in 2 mL DMEM containing 10 % FBS per well of a 6-well plate. The next day, when the cells confluence get 50 %, the medium was changed to Opti-MEM I Reduced Serum Medium (Hyclone, Logan, Utah, USA), and cells were transfected with 10 μL siRNA using 10 μL Lipofectamine 2000 Reagent (Invitrogen). After 4–6 h, medium was replaced by MEM containing 10 % FBS (Hyclone, Logan, Utah, USA). The cells were incubated for 24–48 h. Transfected cells were washed twice with cold PBS (Hyclone, Logan, Utah, USA). At least three independent experiments were performed for each assay, each time with a minimum of n = 4. Opti-MEM I Reduced Serum Medium was used as diluent.

### Western blotting

The whole-cell protein of BHK-21 and C2C12 were collected using RIPA and PMSF, and conserved at −20 °C. The concentration of protein was detected using the bicinchoninic acid (BCA) method (Beyotime Biotechnology, Jiangsu, China). Subsequently, samples were separated by SDS–PAGE (10 %) and electroblotted onto polyvinylidene fluoride (PVDF) membrane (Millipore, USA). Membrane was blocked with 1× TBST supplemented with 5 % skimmed milk powder (servicebio, Wuhan, China). Membranes were incubated with the primary antibody, anti-SMG5 (P-14) (goat. #SC50980, Santa Cruz Biotechnology, Inc.). For normalization of the results, membranes were reblotted for *β*-actin (anti-*β-*actin, servicebio, Wuhan). Proteins were detected with secondary antibody (HRP-rabbit, anti-goat, 1:1000; boster, Wuhan, China). For the TBL2 and GADD45B western blotting analysis, the primary antibodies were anti-TBL2 (L-15) (goat. #SC-104692, Santa Cruz Biotechnology, Inc.), and anti-GADD45B (rabbit, #ab128920, abcam, Inc.). Bands on the X-ray films were quantified with WCIF ImageJ software for the densitometry analysis.

### Statistical analysis

All data were displayed as the mean ± SD of three independent experiments, in which each assay was performed in triplicate. Statistically significant differences between two groups were determined by Student’s t test. P < 0.05 was considered statistically significant.

## Abbreviations

miR-433: microRNA-433
NMD: nonsense mediated mRNA decay
SMG5: suppressor with morphological effect on genitalia 5
PTC: premature termination codon
TBL2: transducin beta-like protein 2
GADD45B: growth arrest and DNA damage-inducible protein GADD45 beta
UTR: untranslated regions
UPF1: up-framshift 1
SMG7: suppressor with morphological effect on genitalia 7
PP2A: protein phosphatase 2A
PNRC2: prorich nuclear receptor co-activator 2
SFRP2: secreted frizzled-related protein 2
GRB2: growth factor receptor-bound protein 2
CREB1: CAMP-response element binding protein
HDAC6: histone deacetylase 6
